# Impact of a Dengue Outbreak Experience in the Preventive Perceptions of the Community from a Temperate Region: Madeira Island, Portugal

**DOI:** 10.1371/journal.pntd.0003395

**Published:** 2015-03-13

**Authors:** Teresa Nazareth, Carla Alexandra Sousa, Graça Porto, Luzia Gonçalves, Gonçalo Seixas, Luís Antunes, Ana Clara Silva, Rosa Teodósio

**Affiliations:** 1 GABBA Doctoral Program, ICBAS, Abel Salazar Institute for the Biomedical Sciences, University of Porto, Porto, Portugal; 2 Unidade Clínica Tropical, Instituto de Higiene e Medicina Tropical, Universidade Nova de Lisboa, Lisboa, Portugal; 3 Unidade de Parasitologia Médica, Instituto de Higiene e Medicina Tropical, Universidade Nova de Lisboa, Lisboa, Portugal; 4 Unidade de Parasitologia e Microbiologia Médica, Instituto de Higiene e Medicina Tropical, Universidade Nova de Lisboa, Lisboa, Portugal; 5 Basic & Clinical Research on Iron Biology, IBMC, Institute for Molecular and Cellular Biology, Porto, Portugal; 6 Unidade de Saúde Pública e Internacional e Bioestatística, Instituto de Higiene e Medicina Tropical, Universidade Nova de Lisboa, Lisboa, Portugal; 7 Centro de Estatística e Aplicações da Universidade de Lisboa (CEAUL), Lisboa, Portugal; 8 Departamento de Saúde, Planeamento e Administração Geral, Instituto de Administração da Saúde e Assuntos Sociais, Funchal, Região Autónoma da Madeira, Portugal; 9 DROTA—Direção de Serviços de Informação Geográfica e Cadastro, Direção Regional de Ordenamento de Território e Ambiente, Funchal, Região Autónoma da Madeira, Portugal; 10 Centro de Malária e Doenças Tropicais, Instituto de Higiene e Medicina Tropical, Universidade Nova de Lisboa, Lisboa, Portugal; Common Heritage Foundation, NIGERIA

## Abstract

The ability to effectively modify behaviours is increasingly relevant to attain and maintain a good health status. Current behaviour-change models and theories present two main approaches for (healthier) decision-making: one analytical/logical, and one experiential/emotional/intuitive. Therefore, to achieve an integral and dynamic understanding of the public perceptions both approaches should be considered: community surveys should measure cognitive understanding of health-risk contexts, and also explore how past experiences affect this understanding. In 2011, community perceptions regarding domestic source reduction were assessed in Madeira Island. After Madeira’s first dengue outbreak (2012) a unique opportunity to compare perceptions before and after the outbreak-experience occurred. This was the aim of this study, which constituted the first report on the effect of an outbreak experience on community perceptions regarding a specific vector-borne disease. A cross-sectional survey was performed within female residents at the most *aegypti*-infested areas. Perceptions regarding domestic source reduction were assessed according to the Essential Perception (EP)-analysis tool. A matching process paired individuals from studies performed before and after the outbreak, ensuring homogeneity in six determinant variables. After the outbreak, there were more female residents who assimilated the concepts considered to be essential to understand the proposed behaviour. Nevertheless, no significant difference was observed in the number of female residents who achieved the defined ‘minimal understanding’’. Moreover, most of the population (95.5%) still believed at least in one of the identified myths. After the outbreak some myths disappeared and others appeared. The present study quantified and explored how the experience of an outbreak influenced the perception regarding a dengue-preventive behaviour. The outbreak experience surprisingly led to the appearance of new myths within the population, apart from the expected increase of relevant concepts’ assimilation. Monitoring public perceptions is therefore crucial to make preventing dengue campaigns updated and worthy.

## Author Summary

Since there is no vaccine or treatment available for dengue fever, its prevention relies on community participation. Residents are asked to remove from their houses and gardens all receptacles where mosquitoes can breed. Exploring the public perception regarding dengue prevention is crucial for detecting obstacles to their participation in the proposed preventive activities. The authors explored and compared the community's perceptions before and after the first dengue outbreak in Madeira Island. For the first time it was possible to study the effect of a dengue outbreak in the public perceptions regarding its prevention. After the dengue outbreak, the authors found an improvement in the perception of the community. However, even after experiencing an outbreak, the majority of the residents still did not understand their role in the dengue prevention and, thus were not ready to adhere to it. Moreover, the authors also observed some new myths within the community after the outbreak (which were not present before the outbreak). The improvement of community perceptions was expected. However this search also revealed that this experience can surprisingly promote the emergence of new myths which may hamper the community engagement in the dengue prevention.

## Introduction

Most of the 2011 worldwide major causes of death (MCD), rely on behaviour changes for their prevention [1]. Increasing physical activity, fruits/vegetables intake, hand-washing, use of condoms, and decreasing not only fat, salt and sugar intake but also smoking habits, are crucial in the control of heart disease (1^st^ MCD), stroke (2^nd^ MCD), chronic obstructive lung disease (4^th^ MCD), diarrhoea (5^th^ MCD), HIV (6^th^ MCD), or diabetes (8^th^ MCD). Behaviour changes are increasingly relevant to attain and maintain a good health status, especially when facing health threats for which there is no efficient or timely treatment. This is the case for dengue fever that such as other mosquito-borne diseases, requires a good compliance to certain preventive, protective or therapeutic actions. Moreover, since there is no vaccine or treatment for dengue fever, neither 100% effective insecticides, community behaviour has a huge impact on its prevention and control [2].

It is still not widely understood how to effectively promote behaviour changes [3]. During several decades, many behaviour impact campaigns were shown to be fruitless. In the past 50 years, literature extensively presented theoretical models which tried to clarify cognitive ways of (healthier) behaviour acquisition [4,5,6,7,8,9]. Recently, the concept of ‘past experiences’ was stated as being crucial in determining (healthier) decision-making. Many authors claimed that, due to the type of emotions and intuition that they produced, ‘past experiences’ could strongly encourage or discourage a particular action [6,7,8,9,10].

Altogether, these contributions seem to present two different approaches by which humans perceive decision-making and then make decisions: one analytical and one experiential [11]. In order to improve the efficacy of the behaviour-promoting messages, the authors firmly suggested that messages should be not only meaningful but also emotionally adequate for the targeted community. This way, the assessment of community’s cognitive and emotional perceptions, is hence useful in the guiding of effective health-seeking messages. However, few studies explored emotional experience-driven perceptions but rather frequently only focused on the assessment of the cognitive ones [12].

Some evidence suggested that experience can influence public perceptions and reactions in two ways [13]. In one aspect, it can over-estimate the risk perception [10,13] (i.e. alert-feeling, referred to as ‘availability bias’ [14]) and consequently promote protective/preventive actions. It can also underestimate the risk perception [12,15] (i.e. habituation effect also called ‘gambler’s fallacy’[14]) which can discourage protective/preventive actions. Only few studies have explored this issue in real situations. Besides the scientific interest of scrutinizing the complex process of (healthier) decision-making, the monitoring of public perceptions and behaviours contributes to the continuous and adequate update of the behaviour-promoting messages concerning their (rational and emotional) content. This is the case of any chronic and endemic disease, where the (health) risk is maintained during time such as dengue epidemic and endemic areas [12].

In 2005, a dengue vector species, *Aedes aegypti* was reported in Madeira archipelago. In 2012, the first dengue outbreak was recorded in the territory [16]. Community perception regarding preventive behaviours (domestic source reduction) was assessed and described in details by the current investigators, before the outbreak had been declared [17]. At the end of the outbreak, a unique opportunity to explore and compare community perception before and after the outbreak appeared. The aim of this study was thus to re-assess community perceptions regarding the same preventive behaviour (domestic source reduction) just after the dengue outbreak in order to compare how it has altered with the outbreak experience. This constitutes to our knowledge the first report of this kind describing the effect of an outbreak experience on community perceptions regarding a specific vector-borne disease.

## Methods

As subsequently explained, methodology of the present survey (herein stated as POST-outbreak study) followed as much as possible the methodology used in the prior-to-the outbreak survey (herein mentioned as PRE-outbreak study) [17]. This ensured an accurate comparison between public perceptions before and after the dengue outbreak in Madeira Island.

Therefore, the tool used in the assessment of the community perceptions was maintained, *i*.*e*., the ‘Essential-Perception analysis’ (see sub-section of the same name). However, since the outbreak was not planed ahead nor predicted, in the POST-outbreak survey was not possible to reproduce exactly the same methodology used before the outbreak. Due to ethic, time and logistic constrains implicit in the preparation of this survey during the outbreak and in its implementation just after it, adjustments in the size of the studied sample and in the sampling methodology, were mandatory to make the POST-outbreak survey possible. The introduced alterations were sample size reduction (through rural and male residents’ exclusion) and intentional sample selection instead of the previous random one. These alterations are explained in detail in ‘Studied population’ sub-section. Finally a matching process was developed in order to overcome those constrains and guarantee an unbiased comparison between the two studies despite their differences in sampling methodology. For that, populations surveyed in both PRE/POST-outbreak studies and who had fully completed the questionnaires were scored according to the perceptions demonstrated (for EP-Score calculation) and marked according to the six socio-demographic characteristics (for the matching process). After this, populations were matched according to critical socio-demographic variables, as described in sub-section ‘Matching Process’ and EP-score was compared within matched pairs. Individuals who presented missing questions were excluded from the analysis.

### Studied Population

**Fig 1:**
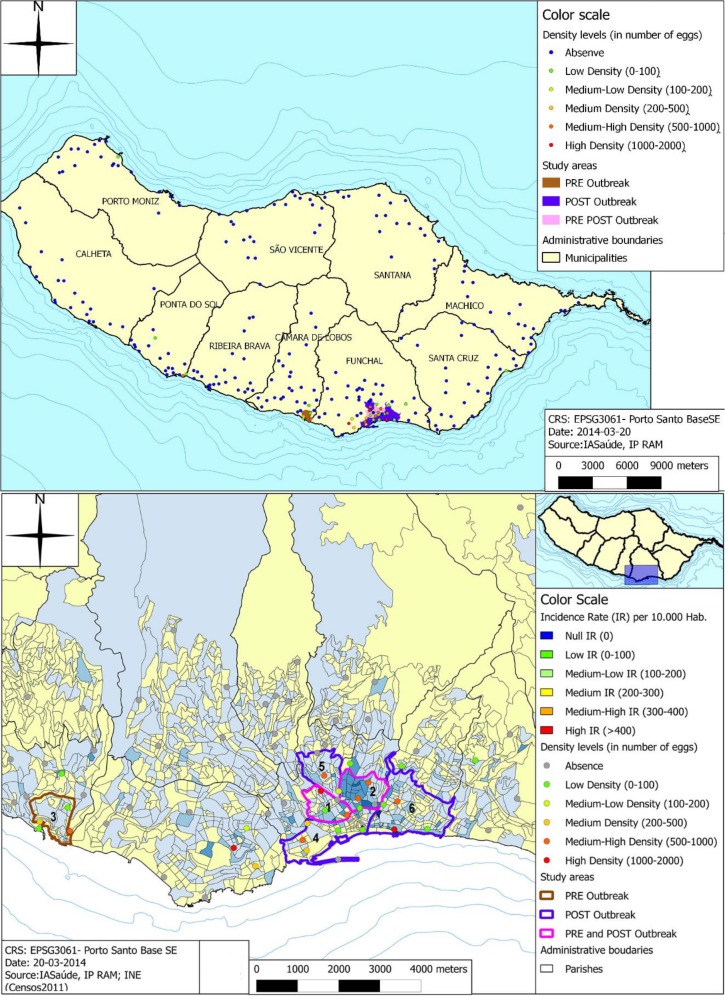
Study areas from PRE-outbreak and POST-outbreak studies. Fig. 1 (upper) shows *Aedes aegypti’*s distribution (2011) resulted from the Island-wide transversal entomological survey using ovitraps. Study areas of both PRE-outbreak and POST-outbreak studies were also described. Black administrative boundaries described are relative to Island Counties (or Districts). Pink area corresponds to the area included in both PRE-outbreak and POST-outbreak surveys (it also corresponds to what is defined the ‘urban area’). Brown area corresponds to the area included in PRE-outbreak survey but not in the POST-outbreak one (corresponds to the ‘rural area’). Blue area corresponds to the area included only in the POST-outbreak survey. Fig. 1 (**bottom**) detail of the previous maps focusing on the density levels based on the number of eggs founded and on the incidence rates of the study areas. Administrative boundaries described are relative to parishes (or ‘Municipalities’) Pink area corresponds the area included in both PRE-outbreak and POST-outbreak surveys. Brown area corresponds to the area included in PRE-outbreak survey but not in the POST-outbreak one. Blue area corresponds to the area included only in the POST-outbreak survey. Numbers represent the part of each municipality covered in the study areas: 1- São Pedro; 2- Santa Luzia;3-Câmara de Lobos; 4- Sé; 5- Imaculado Coração e Maria; 6- Santa Maria Maior.

Out of the several municipalities which were covered by the PRE-outbreak study area, only some were selected to be included in this POST-outbreak study (Fig. 1). In order to decrease the sample size, ‘Câmara de Lobos’ was excluded since it was the sole rural municipality. Facing the impossibility of including both urban and rural municipalities, the urban ones were preferred based on two main reasons: (i) they presented a dengue incidence rate greater than 200 during the outbreak (S1 Fig.); and (ii) they comprise the capital city of the archipelago, Funchal, and thus an important point of *aegypti*-dispersion. Part of the Funchal municipalities: ‘Sé’, ‘Santa Maria Maior’ and ‘Imaculado Coração de Maria’, were also included in the POST-outbreak study area besides those considered in the PRE-outbreak study (‘São Pedro’ and ‘Santa Luzia’). These extra-included area were also covered by the 2012’s most *aegypti*-infested area (presenting a density level of 31% or over along the year), thus ensuring a homogeneous level of natural exposure to the *A. aegypti* among the studied residents [18]. The geographic area covered in the present study will be mentioned as ‘Extended-AEGYPTI area’ and consists, thus, in part of five Funchal’s municipalities from the 2012 most *aegypti*-infested area.

Due to the mentioned unfeasibility to include a representative sample of the resident population of the study area, an intentional sample of exclusively female subjects who lived in the study area—‘Extended-AEGYPTI area’ aged 18 years old or over and who didn’t integrate the previous PRE-outbreak survey was selected from customers of central hairdressers and pharmacies, placed in the selected area. The women selection was based on three main reasons: (i) before the outbreak women were significantly less aware of domestic source reduction than men (S2 Fig. and S1 Table); (ii) women are the majority within the studied population [19]; (iii) women above 15 years-old were the age/gender-group more affected by the disease during the outbreak [20]; and (iv) culturally, in Madeira Island, women are more related to the main dengue-preventive behaviour proposed than men do (see details about the behaviour proposed in ‘Essential-Perception’ subsection). All women who entered in the establishment and who met the inclusion criteria were invited to participate.

The type of establishment were chosen in order to allow the study to cover the most possible heterogeneous women sample, in what concerns their age groups, education levels and socio-economic background. In order to stimulate participation of women from all the included municipalities, two central establishments of each service were chosen to participate in the study according to their convenient geographical location, being one placed in the east and the other in the west region of the studied area.

The study area has a population density which can be as high as 1433.5 habitants per square kilometers [21]. The sample size was calculated using Epitools’ sample size calculators (2014, AusVet Animal Health Services) in order to perform a comparison of two means using the t-test (2-tailed) where a 1 point is relevant in Essential-Perception Score difference (variation between −10, 10) [22]. The sample size calculation considered a 95% confidence level, a power of 80% and a pooled variance equal to 10 (S = √10 = 3.16). The obtained n was 157 (S2 Table). Finally, this sample size was inflated in 30% to account for incomplete interviews.

### Questionnaire

A cross-sectional survey was performed to assess residents’ perceptions through face-to-face interviews. Before data collection, establishments’ managers/participants gave their written/oral informed consent respectively. Previous to the beginning of this survey, the questionnaire was pre-tested in a non-selected establishment placed in the selected area. During the interview, a questionnaire comprising 21 questions was applied, covering dengue-preventive issues and personal-socio-demographic characteristics. In agreement with what was inquired in the PRE-outbreak study, questionnaire covered five main topics: ‘Medical Importance’ (two questions), ‘Local Context’ (two questions), ‘Domestic Attribute’ (three questions), ‘Mosquito Breeding’ (three questions) and ‘Control Measures’ (three questions) [17]. Besides the variables ‘gender’, ‘education level’, ‘age group’, and ‘geographical area’, two other variables were assessed: ‘travels to dengue endemic countries (DEC)’ which measures who had already been to any dengue endemic country and ‘admitted mosquito exposure (AME)’ which measures who recognized to had been bitten by mosquitoes. The survey was performed by trained personnel from the local health authority from 22^nd^ of March until 16^th^ of April, 2013. In each establishment (pharmacies/hairdressers), interviews were performed during a Monday-to-Saturday week, between 9am and 7pm (according to establishments’ opening hours) The study was approved by Instituto de Higiene e Medicina Tropical Ethics Committee, Instituto de Higiene e Medicina Tropical, Universidade Nova de Lisboa, Lisbon (reference: 09-2013-TD).

### Matching Process

Populations studied in both PRE/POST-outbreak surveys were matched into pairs, ensuring homogeneity in six critical socio-demographic variables. Resulting matching population comprised thus pairs of individuals composed of an individual from the PRE-outbreak study and an individual from the POST-outbreak study with equivalent personal-socio-demographic characteristics. Matching pairs of individuals were equal in (or “blocked” on) gender, education level, age group, geographical area, travels to DEC and AME variables, already shown to be determinants to the individual perception [17]. This sampling methodology can also be called as randomized block design, and the latter variables as blocking factors [23].

For comparative purposes, the criteria ‘geographic area’ was applied in two different ways, generating two different matching approaches. In one matching approach, herein called ‘adjusted matching’, the ‘geographic area’ criteria distinguished only residents living in urban areas from residents living in rural areas. According to this criteria, ‘Câmara de Lobos’ (covered exclusively in PRE-outbreak study area) was the sole rural municipality. In the other matching approach, herein called ‘restricted matching’, the geographic criteria besides the previous distinction between urban and rural areas also differentiated urban municipalities covered in both PRE-outbreak and POST-outbreak studies (‘Santa Luzia’ and ‘São Pedro’) from the remaining urban ones which were exclusively included in the POST-outbreak study (‘Sé’, ‘Santa Maria Maior’ and ‘Imaculado Coração de Maria’). The other criteria (gender, education level, age group, travels to DEC and AME) were strictly applied in both matching approaches, *i*.*e*. only individuals who were equal in this variables were matched.

Both matching procedures were built in Excel (Microsoft Office, Windows 8), and guaranteed that individuals were randomly selected within those that were personal-socio-demographically equivalent. Moreover, matching procedures were optimized in order to re-include all the non-selected individuals in the subsequent matching rounds.

### Essential-Perception Analysis (Perception Evaluation)

The assessment of the community perception was performed using the Essential-Perception analysis (EP-analysis), as described below.

Essential-Perception analysis assesses community perception regarding a particular behavioural proposal: the domestic *aegypti’s* source reduction, considered the most critical dengue-preventive practice by the World Health Organization [24]. This corresponds to the elimination (emptying, covering or removing) of water-containers present inside or around residential buildings. The EP-analysis’ considers ten essential concepts which assimilation by individuals was revealed to be determinant to the performance of the proposed behaviour (Table 1) [17]. Essential-Perception analysis allows the characterization and estimation of the community’s perceptions through four different approaches, all of them used here: (i) score of Essential-Perception, (ii) concept assimilation, (iii) topic understanding and (iv) myth identification and estimation. The first measures the number of concepts that were assimilated (out of those defined to be ‘essential’) and thus how far-off is the studied population from achieving the complete ‘Essential Perception’ (EP-Score = 10). The second describes how much those ‘essential’ concepts were assimilated or not-assimilated by the community. The third, organizes the ‘essential concepts’ in topics and describes how topics are/not being understood. Residents who have acknowledged both topic-related concepts are according to this tool considered to have ‘completely understood the topic’, the acknowledgement of only one out of the two topic-related concepts is considered as a ‘partial understanding of the topic’, and residents who did not perceive any of the two topic-related concepts are considered to have ‘not understood the topic’. Finally the fourth, by analysing the concept assimilation, identifies erroneous beliefs which may persist in the community (herein mentioned as ‘myths’) and estimates their putative frequency in the studied population (see example in S3 Fig.).

**Table 1:** List of ten concepts defined as essential within Essential Perception-analysis.

Essential Topic	Essential Concepts
Medical Importance (MI)	MI1-concept- Recognition of the transmission of disease through mosquitoes (bite)
	MI2-concept—Recognition of the one example of mosquito-borne diseases
Local Context (LC)	LC1-concept—Recognition of the presence of vector-mosquitoes in their own residential area
	LC2-concept—Recognition of the high possibility of a dengue outbreak in Madeira
Domestic Attribute (DA)	DA1-concept—Recognition of the eventuality of indoor mosquito-breeding
	DA2-concept—Recognition of the impact of domestic vector control
Mosquito Breeding (MB)	MB1-concept—Recognition of the role of water-containers as breeding contributors
	MB2-concept—Recognition of the false role of ‘pets’ or ‘food debris’ as breeding contributors
Control Measures (CM)	CM1-concept—Recognition of source reduction as an effective domestic *aegypti*-control measure
	CM2-concept—Recognition of ‘insecticide application’ or ‘use of a flyswatter’ as ineffective measures for the domestic *aegypti*-control’

### Statistical Analysis

All collected information was introduced and records were double-checked. Statistical analysis was performed using Statistical Package for Social Sciences 19.0 (SPSS, Inc., Chicago, IL, USA). Answers obtained from the questionnaires were re-coded to obtain other categorical variables implicit in the EP-analysis. The personal-socio-demographic feature of the studied population presented in Table 2 was described in what concerns the gender, age group, education level, municipal division, travels to dengue endemic countries (DEC) and AME. The age groups were categorized in ten-year intervals and the education level was divided into five categories starting from ‘never studied’ until ‘upper graduation’. This categorization allow that groups were similar in number of individuals.

**Table 2:** Personal-socio-demographic feature description of Total and Paired samples in both PRE-outbreak and POST-outbreak studies (adjusted matching).

	Total Sample	Total Sample	Paired Sample^•^(No. of pairs = 90)
	PRE-out. Study (n = 1145) ^ⱡ^	POST-out. study (n = 154)	
**Gender**			
Female	466 (40.7%)	154 (100%)	90 (100%)
Male	679 (59.3%)	-	-
**Education level (years)**			
Never studied (0)	69 (6.0%)	44 (28.6%)	12 (13.3%)
Fourth Grade (4)	438 (38.3%)	31 (20.1%)	24 (26.7%)
Ninth Grade (9)	254 (22.2%)	30 (19.5%)	18 (20.02%)
High School (12)	204 (17.8%)	43 (27.9%)	31 (34.4%)
Upper Education (+12)	180 (15.7%)	6 (3.9%)	5 (5.6%)
**Age groups (years)**			
25 or younger	152 (13.3%)	7 (4.5%)	4 (4.4%)
26–35	157 (13.7%)	19 (12.3%)	9 (10.0%)
36–45	186 (16.2%)	24 (15.6%)	17 (18.9%)
46–55	198 (17.3%)	40 (26.0%)	21 (23.3)
56–65	170 (14.8%)	36 (23.4%)	19 (21.1%)
66–75	160 (14.0%)	21 (13.6%)	14 (15.6%)
76 or older	122 (10.7%)	7 (4.5%)	6 (6.7%)
**Municipal Division**			
Funchal	666 (58.2%)^+^	154 (100.0%)‘	90 (100.0%)‘
Câmara de Lobos	479 (41.8%)	-	-
**Travelled to DEC** ^**1**^			
yes	863 (75.4%)	89 (57.8%)	62 (68.9%)
no	282 (24.6%)	65 (42.2%)	28 (31.1%)
**Admitted MQ exposure (AME)** ^**2**^			
yes	286 (25.0%)	46 (29.9%)	22 (24.4%)
No	859 (75.0%)	108 (70.1%)	68 (75.6%)

Since paired sample from both studies were equivalent in these six variables their socio-demographic characteristics are equal (presented as ‘Paired sample’).

^1^ Distinguish those that have/not travelled to dengue endemic countries (at least once)

^2^ Reflects those who admitted/not to had been bitten by mosquitoes

^ⱡ^ Individuals that were scored regarding the 13 questions for perception assessment and that also have answered to the personal-socio-demographic questions implicated in the matching process

^•^Adjusted matching

* Differences resulted from the adjustment done in in this matching process

^+^Including ‘Santa Luzia’ and ‘São Pedro’ municipalities

‘Including ‘Santa Luzia’, ‘São Pedro’, ‘Sé’, ‘Imaculado Coração de Maria’ and ‘Santa Maria Maior’ municipalities

Comparison of the two urban municipalities covered in both PRE-outbreak and POST-outbreak studies (‘Santa Luzia’ and ‘São Pedro’) confirmed a *priori* the validity of the criteria ‘geographic area’ in the restricted matching. In this matching those municipalities were considered equivalent. S3 Table shows that despite the previous observed differences observed in their EP-score level, when “blocking” the education level there are no significant differences between the two municipalities.

Analysis of the demographic data of the extra-included areas compared the new added municipalities (‘Sé’, ‘Santa Maria Maior’ and ‘Imaculado Coração de Maria’) and the previously studied (‘Santa Luzia’ and ‘São Pedro’). Comparison is presented in S4 Table showing that there were no relevant differences between them in what concerns the two critical socio-demographic determinants: age group and education level supporting thus a *priori* the validity of the criteria ‘geographic area’ in the adjusted matching.

Comparisons of EP-score medians between populations from PRE/POST-outbreak studies were made using the non-parametric Wilcoxon Test (Table 3), after rejecting the normality of Essential-Perception score difference through Kolmogrov-Smirnov test. Additionally, the number of individuals who achieved an EP-score equal to or higher than seven (EP-score≥7) was calculated in both studies and differences were compared, using the McNemar Test (Table 4). This cut-off was chosen due to lack of subjects that achieved an EP-score equal to ten (EP-score = 10). In order to evaluate the methodology used during the matching process, comparisons between paired and non-paired samples were performed, according to their EP-score and their socio-demographic characteristics. In order to ensure that restricted and adjusted matching sample sizes (n = 47and n = 90) were satisfactory, the power associated to Wilcoxon test (the non-parametric alternative to t-test) was calculated *a posteriori* using free statistical power analysis program, G*Power 3.0 [25].

**Table 3:** The EP-scores from Total and Paired samples of both PRE/POST-outbreak surveys and associations between them.

	n Total ⱡ (matching compatible)	EP-Score medians (P_25_-P_75_) ^+^	n Paired	EP-Score medians (P_25_-P_75_) ^+^	*p* value
**PRE-outbreak survey**	1145^•^	5.0 (3.0–6.0)	90	5.0 (4.0–7.0)	0.952′
				↕	
**POST-outbreak survey**	154	7.0 (5.0–8.0)	90	7.0 (6.0–8.0)	0.073′
			***p* value**	<0.001* ‘’	

+ Weighted Average method

* Wilcoxon test

‘ Mann-Whitney test

‘’ t-test

ⱡ number of individuals compatible for matching *i*.*e*. individuals who were scored regarding the 13 questions for perception assessment and who also have answered to the personal-socio-demographic questions implicated in the matching process.

• Out of the 1182 individuals that were scored in the PRE-outbreak study, 37 subjects were not included in the matching process, since they lack critical socio-demographic data

**Table 4:** Evolution of the residents’ groups according to the cut-off: EP-Score ≥ 7 (Before/After the outbreak).

		POST-outbreak survey (paired population)		*p* value
		EP-Score < 7	EP-Score ≥7	
**PRE-outbreak survey (paired population)**	**EP-Score < 7**	27 (=)	38 (↑)	<0.001^+^
	**EP-Score ≥ 7**	4 (↓)	21 (=)	

nTOTAL = 90 pairs

(=) Number of individuals that did not change the EP-Score level compared with its pair

(↓) Number of individuals that have increased the EP-Score level compared with its pair

(↑) Number of individuals that have decreased the EP-Score level compared with its pair

+ McNemar test

## Results

**Fig 2:**
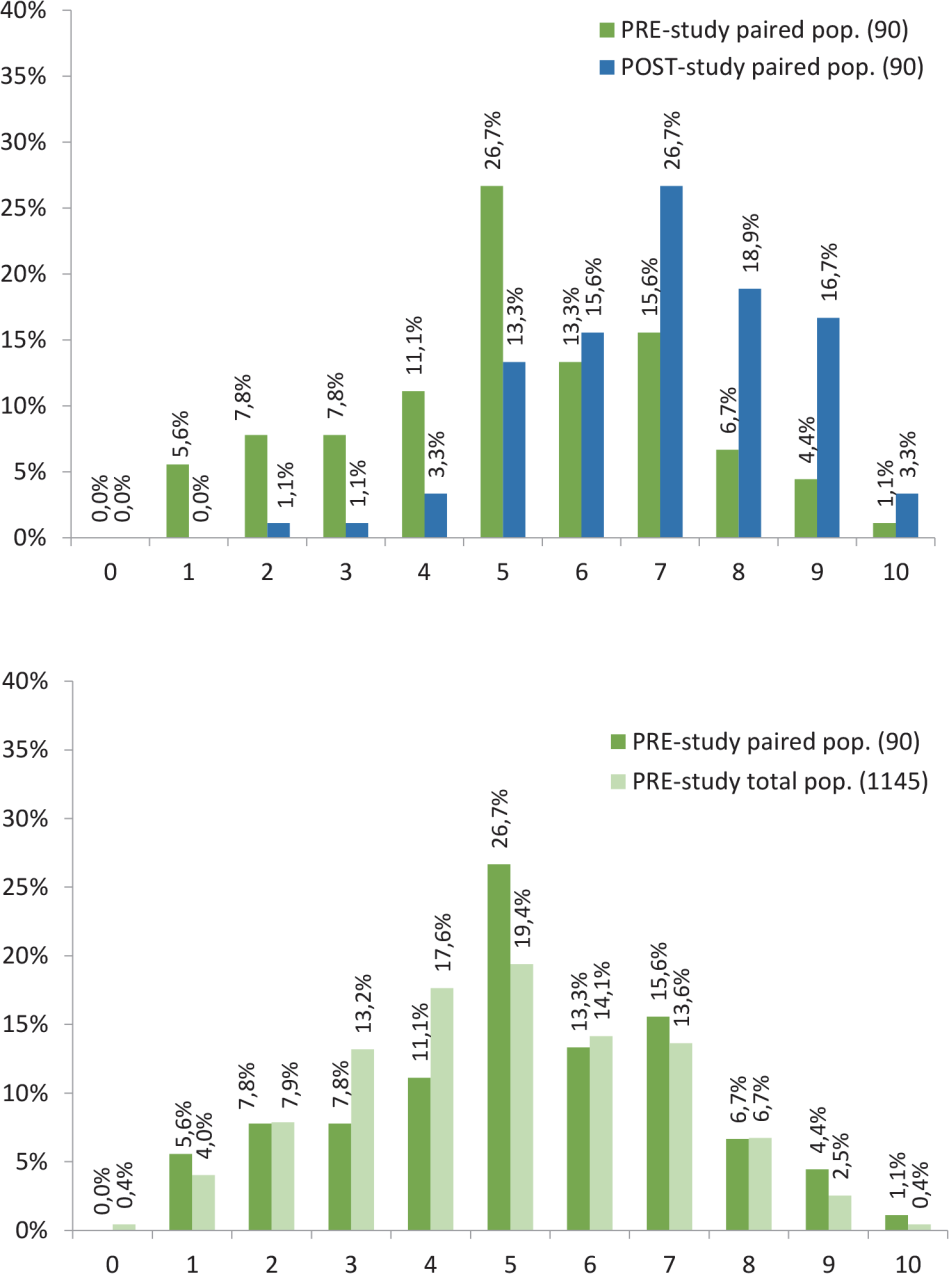
The EP-Score distributions of the paired samples and of the total sample in PRE-outbreak study. Comparison of EP-score distribution from paired samples of both PRE/POST-outbreak studies (upper) and comparison of EP-score distribution from total and paired samples of the PRE-outbreak study (bottom) are presented.

**Fig 3:**
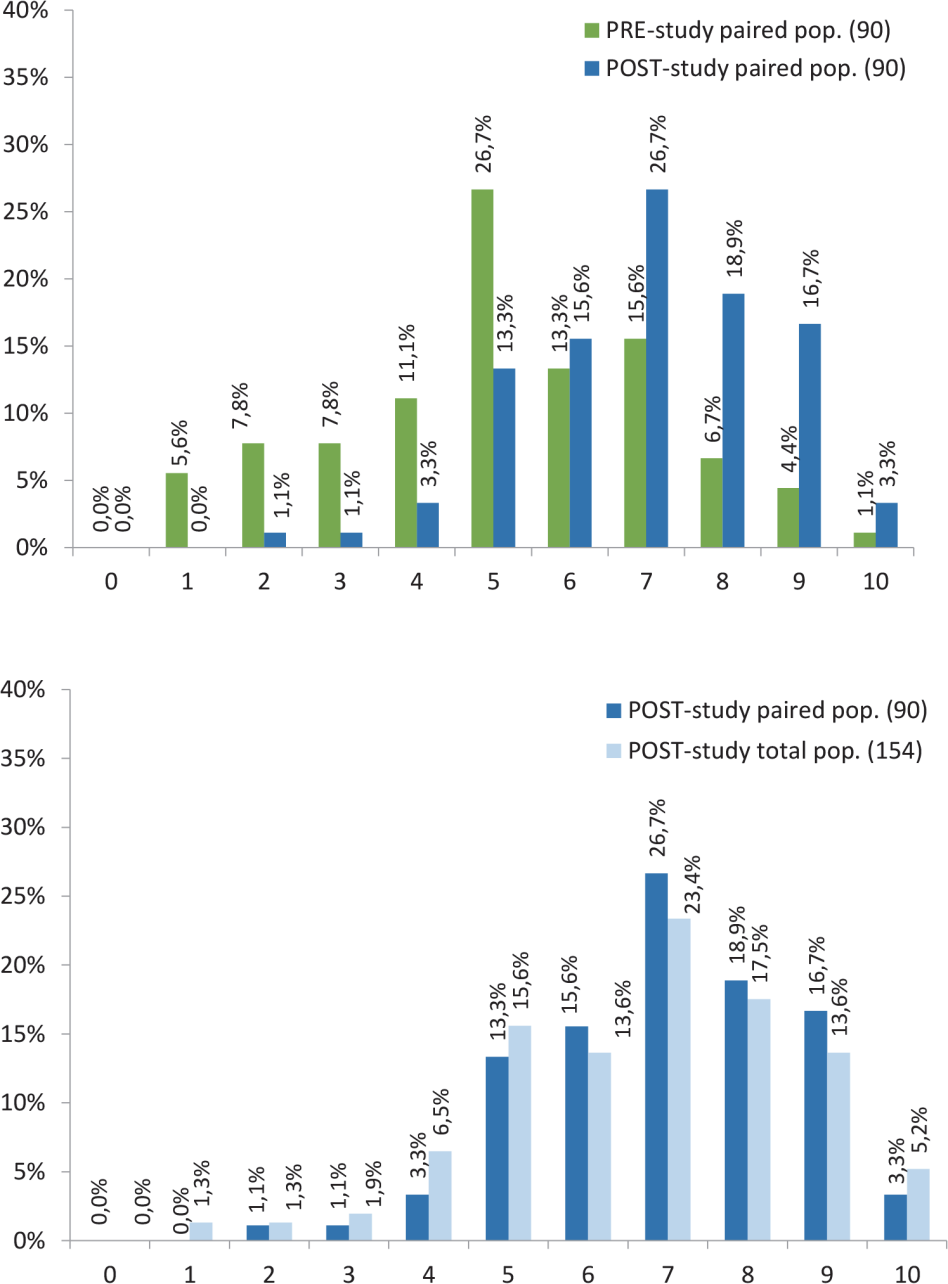
The EP-Score distributions of the paired samples and of the total sample in POST-outbreak study. Comparison of EP-score distribution from paired samples of both PRE/POST-outbreak studies (upper) and comparison of EP-score distribution from total and paired samples of the POST-outbreak study (bottom) are presented.

**Fig 4:**
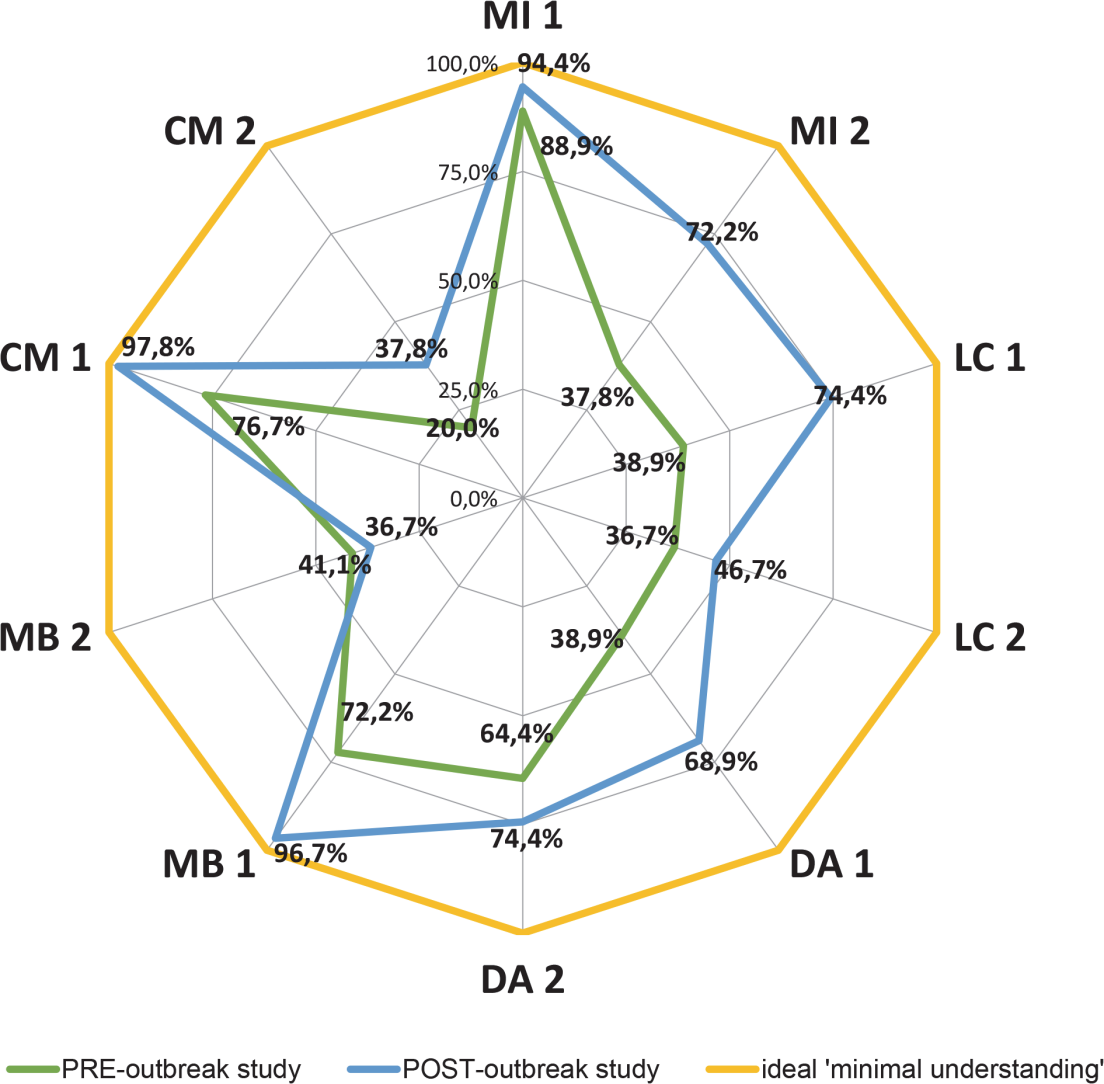
Percentage of residents who have assimilated each Essential concepts in both PRE-outbreak and POST-outbreak studies. For Figure simplification, essential concepts were abbreviated to their name initials: Medical Importance 1 and 2 (MI 1 and MI 2), Local Context 1 and 2 (LC 1 and LC 2), Domestic Attribute 1 and 2 (DA 1 and DA 2), Mosquito Breeding 1 and 2 (MB 1 and MB 2), Control Measures 1 and 2 (CM 1 and CM2).

**Fig 5:**
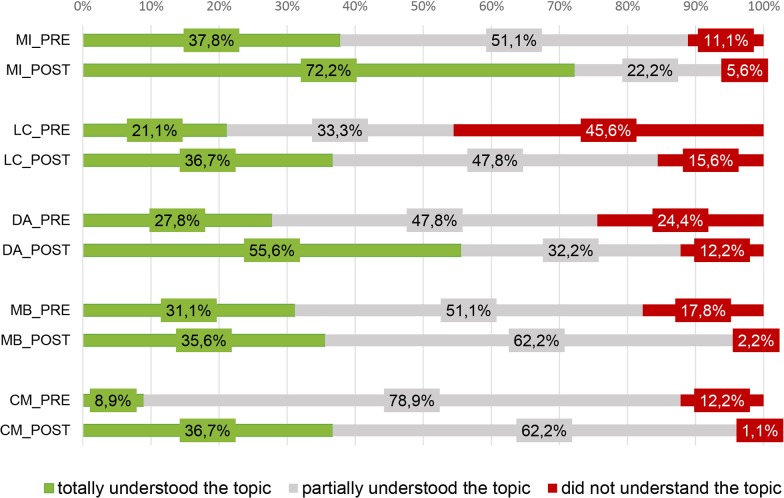
Percentage of residents who have ‘understood’, ‘partially understood’ or ‘not understood’ each studied topic. For Figure simplification, topics were abbreviated to their name initials: Medical Importance (MI), Local Context (LC), Domestic Attribute (DA), Mosquito Breeding (MB), Control Measures (CM) ‘PRE’ and ‘POST’ represents PRE-outbreak study and POST-outbreak study.

A total of 154 female Extended-AEGYPTI residents answered the complete questionnaire in the present POST-outbreak survey. A total of 90 pairs resulted from the adjusted matching between 154 female from the POST-outbreak survey and 1145 subjects who participated in the PRE-outbreak study. Each pair composed of an individual from the PRE-outbreak study and an individual from the POST-outbreak study with equivalent personal-socio-demographic characteristics. Nine individuals out of those surveyed had dengue and five were paired. The personal-socio-demographic feature of the studied sample populations is described in Table 2. When not mentioning subsequent paragraphs as well as the data presented in Figs. 2, 3, 4 and 5, and Tables 2, 3, 4 and 5 present results from the adjusted matching.

**Table 5:** Alleged myths in both PRE-outbreak and POST-outbreak studies and respective frequencies The myths which were derived from PRE-outbreak study were re-numbered.

			PRE-outbreak study	POST-outbreak study	Difference
Essential Topics	Old / New No.	Alleged Myths	n (%)	n (%)	
Medical Importance	Myth 1	‘Mosquitoes do not transmit diseases’	10 (11.1)	5 (5.6)	↓
	Myth 2	‘Mosquitoes only cause mild clinical consequences such as allergies, fever, etc.’	46 (51.1)	20 (22.2)	↓↓↓
Local Context	Myths 3and 4	‘Dengue is not a mosquito-borne disease’ and/or “Dengue only occur in tropical/non-developed countries”	14 (15.6)		(disappeared)
	Myth 3 and Myth 6a	‘Dengue will not occur again in Madeira, it is very not likely‘		34 (37.8)	(new)
		‘Dengue was, finally, eradicated‘			
	Myths 5and 6	(i) ‘Since I do not feel the byte, I am not at risk of being bitten/infected’; (ii) “Mosquitoes are allocated in a specific area and are not able to spread through my municipality’	14 (15.6)	9 (10.0)	↓
	Myths 4 and 5				
	Myth 7	‘Madeira’s residents are not at risk‘	41 (45.6)		(disappeared)
	Myth 6b	‘Dengue/*A*. *aegypti* was, finally, eradicated‘		14 (15.6)	(new)
Domestic Attribute	Myth 7(myth 8)	‘Local health authorities are the key intervenient in the domestic control of mosquitoes‘	10 (11.1)	12 (13.3)	=
	Myth 8* (myth 9)	‘Insecticides or other protective measures can control mosquitoes‘	33 (36.7) *	17 (18.9) *	↓↓
	Myth 9(myth 10)	‘I am (Community is) not an intervenient in the *aegypti*-control‘	22 (24.4)	11 (12.2)	↓↓
Mosquito Breeding	Myths 10 and 11 (myths 11 and 12)	‘Clean houses or houses without animals do not have mosquitoes’ and/or ‘Clean people have nothing to do concerning the control of mosquitoes’	53 (58.9)	57 (63.3)	=
Control Measures	Myth 12* (myth 13)	‘By the usage of insecticides and/or flyswatter, I am already contributing to the *aegypti*-control‘	72 (80.0) *	56 (62.2) *	↓↓

*Myths 8 and 12 cover the same idea, there were a total of 67.0% of the residents who believed in one or the other myth, i.e. who felt that by the usage of protective measures, they are already contributing to the aegypti-control.

(↓) Differences of 5–10 percentage points

(↓↓) Differences of 10–20 percentage points

(↓↓↓) Differences of more than 20 percentage points

(=) Differences of less than 5 percentage points

### Essential-Perception Analysis

#### Score of Essential Perception (EP-score)

Altogether Figs. 2 and 3 represent the EP-score distribution of four samples: PRE-outbreak study’s total subjects (n = 1145), PRE-outbreak study’s paired subjects (n = 90), POST-outbreak study’s total subjects (n = 154) and POST-outbreak study’s paired subjects (n = 90). In upper part of Figs. 2 and 3 is presented the EP-score distribution from both paired subjects (from PRE-outbreak and POST-outbreak surveys) revealing an increase in the EP-score level after the outbreak. The graph at the bottom of the latter figures show the EP-score distribution from total and paired populations in PRE-outbreak and POST-outbreak studies (respectively) revealing a similar EP-score distribution within the paired and the respective total population. Detailed information is presented subsequently in the ‘statistical analysis’ results sub-section.

#### Concept assimilation

When comparing the observed concept assimilation in both studies, the POST-outbreak study had more individuals who assimilated each of the essential concepts (Fig. 4). The percentage of female residents considering ‘the existence of mosquito-borne diseases’ (MI2-concept), ‘the presence of vector species in their residential area’ (LC1-concept) and that ‘mosquitoes can breed inside houses’ (DA1-concept) almost doubled, from 37.8% to 72.2%, from 38.9% to 74.4% and from 38.9% to 68.9%, respectively. Regarding the remaining essential concepts, they also increased after the outbreak in terms of the percentage of individuals that have acknowledged them, with the exception of the MB2-concept that slightly decreased. Overall, following the experience of a dengue outbreak, almost all the respondents (94.4%, 96.7% and 97.8%) believed that ‘mosquitoes can transmit diseases’ (MI1-concept), recognized ‘water as a mosquito breeding inducers’ (MB1-concept) and referred to ‘the reduction of breeding sites as being a (fairly/very/extremely) effective measure in the control of mosquitoes’ (CM1-concept). In contrast to, there were some essential concepts which remained unknown for the majority of the studied individuals. These were the ‘Local Context 2’, ‘Mosquito Breeding 2’ and ‘Control Measures 2’ which are also the less acknowledged essential concepts. In fact, only 46.7% believed that ‘there is a high possibility for dengue (re-)emergence in Madeira’ (LC2-concept), merely 38.9% correctly admitted to the ‘false role of pets and food debris in the mosquito breeding’ (MB2-concept) and only 37.8% did not identify ‘the use of a flyswatter or indoor insecticide spraying, as effective for *aegypti*-control’ (CM2-concept).

#### Topic understanding

Topic understanding clearly improved after the outbreak (Fig. 5). In general, the percentage of those who had totally understood each topic increased and the percentage of those who did not completely understand each of topic decreased. ‘Medical Importance’ and ‘Domestic Attribute’ topics became completely understood by the majority of the female Extended-AEGYPTI residents (72.2% and 55.6%). Even after the noticeable increase of people that had totally understood the topics ‘Local Risk’, ‘Mosquito Breeding’ and ‘Control Measures’, the majority of the studied residents still did not understand, or only partially understood them. Similar to the PRE-outbreak study, the ‘Local Context’ topic in the POST-outbreak study had the highest proportion of respondents who disregarded both topic-related concepts.

#### Myth identification and estimation

Based on the thirteen myths that were identified in the PRE-outbreak study, an updated list is suggested in Table 5 which includes new myths identified after the outbreak. The frequency of each believed myth were (re-)calculated in S5 Table and are also presented in Table 5. Out of the thirteen alleged myths identified in the PRE-outbreak study, some had most likely disappeared after the outbreak. This was what happened with the myths: «dengue is not a mosquito-borne disease» or «dengue only occur in tropical/non-developed countries» (Table 5). However, new beliefs emerged after the end of the outbreak, such as the idea that ‘Madeira is protected from a second dengue outbreak’ (alleged myths 3, 6a and 6b). Altogether these myths are suggested to be believed by the majority of the female community (53.3%). According to the myth analysis, after the outbreak each female resident believed, on average, in three out of the twelve myths, less than the four myths out of thirteen believed by the average of the residents before the outbreak. Most of them believed at least in one myth either before or after the outbreak (98.9% and 96.7%, respectively). After the outbreak, the most disseminated alleged myth were «clean houses or houses without animals do not have mosquitoes» and «by the usage of insecticides and/or flyswatter, I am already contributing to the *aegypti*-control». These myths were respectively found in 62.3% and 61.2% of the paired sample.

### Statistical Analysis (Test Statistics)

Statistical tests were performed in order to explore the differences between medians of the EP-score from studied populations in both PRE/POST-outbreak studies, confirming a significant increase in the EP-Score median after the outbreak (p<0.001, Table 3). An increase in the number of individuals who achieved an EP-score equal to or higher than seven (EP-score≥7) in the POST-study population was also statistically confirmed (p<0.001, Table 4).

#### Confirming validity of the ‘Matching Process’

**Fig 6:**
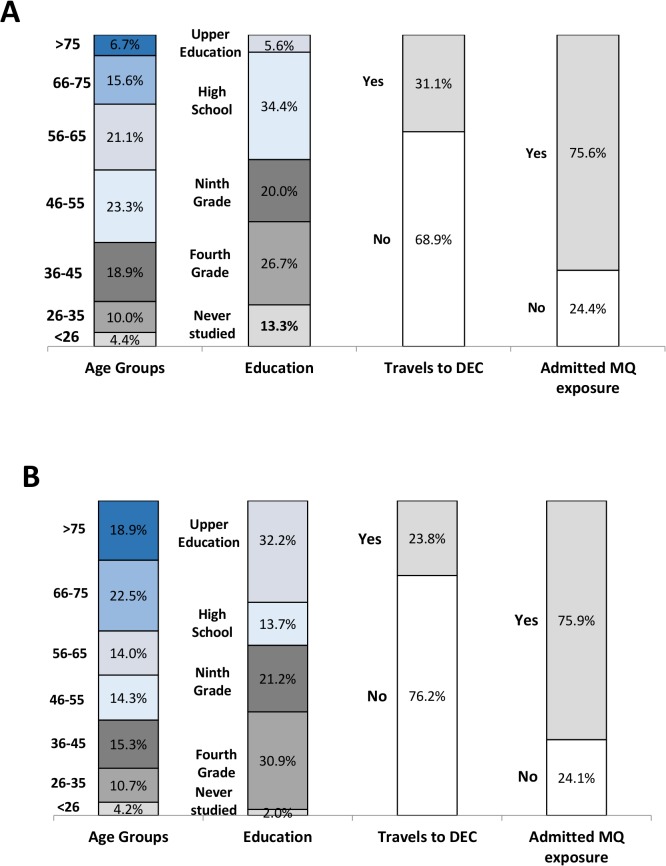
Comparison of personal-socio-demographic data between paired and non-paired samples from PRE-outbreak study. Data from paired sample (A) and data from non-paired sample of the PRE-outbreak study (B) are presented. Age group, Education level, Travels to DEC, and AME (bitten by mosquitoes) variables are presented. Since Gender and geographic are blocked within matching pairs (only female residents from Funchal municipalities were matched) these variables are not presented in these figures.

**Fig 7:**
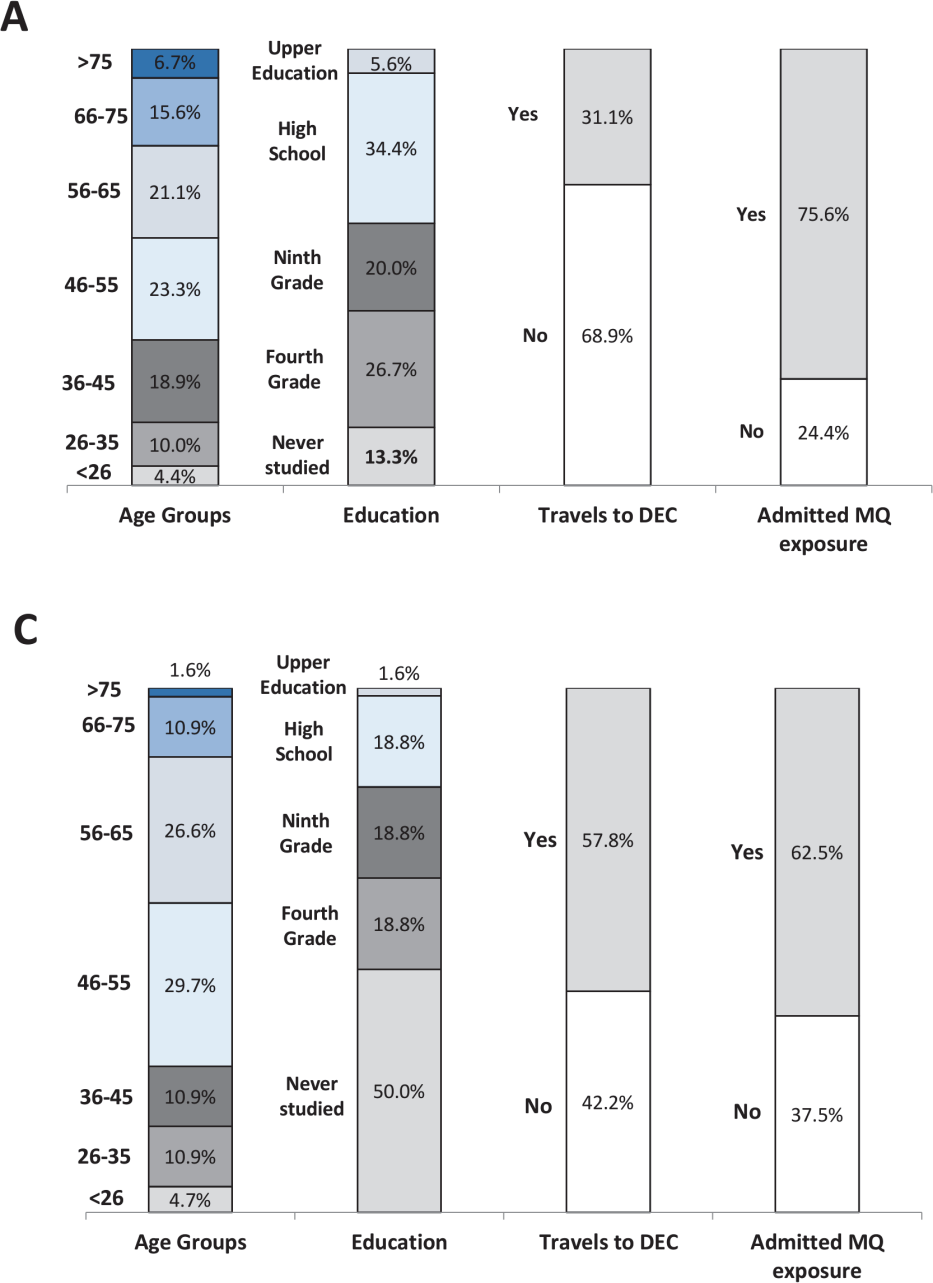
Comparison of personal-socio-demographic data between paired and non-paired samples from POST-outbreak study. Data from paired sample (A) and data from non-paired sample of the POST-outbreak study (C) are presented. Age group, Education level, Travels to DEC, and AME (bitten by mosquitoes) variables are presented. Since Gender and geographic are blocked within matching pairs (only female residents from Funchal municipalities were matched) these variables are not presented in these figures.

The validity of the matching processes was also statistically established. As shown in Table 3 the EP-score from the paired and non-paired samples (in both PRE/POST-outbreak studies) did not change significantly (p>0.05 in both cases). In what concerns the personal-socio-demographic feature, total and paired populations also did not differ expressively (Figs. 6 and 7). Slight differences were detected in proportions of age groups and in high education levels between total populations from PRE-outbreak and POST-outbreak studies.

#### Comparison between restricted and adjusted matching models

The restricted matching resulted in 47 pairs of individuals with equivalent personal-socio-demographic characteristics, being the pairs derived from individuals of the PRE/POST-outbreaks studies performed. The assessed differences in the perception of those surveyed before and after the outbreak, were equivalent to the differences observed, obtained from the comparison of perception of the pairs derived from the adjusted matching. As previously described for the adjusted matching, the EP-score median of the pairs derived from restricted matching has also significantly increased in the POST-outbreak when compared to the PRE-outbreak one (p<0.001). Moreover, the distribution of the EP-score, the concept assimilation and the topic understanding observed for the 47 pairs selected by the restricted matching, were approximately the same that the ones measured for the 90 pairs resulted from the adjusted matching. Additionally, a significant increase of the number of individuals that achieved an EP-score equal to or higher than seven (EP-score≥7) in the POST-study paired population, was also observed when looking at pairs derived from the restricted matching (p<0.001). Table 6 summarizes the data regarding both matching processes, including the power values which were equal in both cases.

**Table 6:** Comparison between restricted and adjusted matching criteria and respective results.

	Restricted Matching	Adjusted Matching
**Study area included**	AEGYPTI area:	Extended-AEGYTI area:
	Part of ‘Santa Luzia’ and ‘São Pedro’ municipalities, corresponding to the 2011-*aegypti*-most infested area	Part of ‘Santa Luzia’, ‘São Pedro’, ‘Imaculado Coração de Maria’, ‘Santa Maria Maior’ and ‘Sé’ municipalities, corresponding to the 2012-*aegypti*-most infested area
**No. of individuals in the POST Total sample** (matching compatible)**	93	154
**No. of pairs PRE/POST** (POST Paired sample)	47	90
**EP-score medians and percentiles**		
PRE-outbreak	5.0 (3.0–6.0)	5.0 (4.0–7.0)
POST outbreak	8.0 (6.0–9.0)	7.0 (6.0–8.0)
**Power of the Wilcoxon test** (used in the comparison between POST/ PRE EP- Score median)	∼1.000	∼1.000

* No main differences observed (S2 Table)

** Individuals that were scored regarding the 13 questions for perception assessment and that also have answered to all the personal-socio-demographic questions implicited in the matching process

## Discussion

In general, the community perception regarding preventive domestic practices improved within female residents of most *aegypti*-infested areas in Madeira Island after they experienced a dengue outbreak. By analysing how and how much assimilation of each 'Essential-concept’ has changed, crucial information can be retrieved regarding people´s perceptions about this experience and their future role in its prevention.

For many Madeira residents, the experience of this dengue outbreak was probably the first contact with a mosquito borne disease (it was the first in almost a hundred years in Europe, [26]. This can explain the observed increase in the assimilation of the idea that ‘mosquitoes can transmit diseases’ (MI1-concept). Moreover, before experiencing the outbreak, the community's worst incident with mosquitoes were allergic reactions, which could be considered as the sole health consequence of mosquito bites. After the outbreak, it was not surprising that the percentage of residents that were aware of ‘the kind of diseases that mosquitoes can transmit (such as dengue, yellow fever and malaria)’ (MI2-concept) almost doubled. Therefore, in the POST-outbreak study there were a higher percentage of people who rightly appraised the impact of mosquitoes in health. Since no fatal cases occurred during the dengue outbreak, some beliefs such as, ‘dengue disease does not kill’ and ‘dengue in Madeira is less aggressive’ may be present in the community. These questions should be clarified within the community due to the possibility of a different virus serotype reaches the Madeira territory, increasing the risk of dengue haemorrhagic cases to occur.

Even though assimilation of both ‘Local Context’ concepts increased after the outbreak, the majority of residents still ignored that ‘there is a high possibility for a (second) dengue outbreak in Madeira’ (LC2-concept). The acknowledgement of this concept was expected to increase after the outbreak, assuming that the previous identified myth which states that ‘Madeira were not at risk of have dengue’ would be opposed with the experience of a dengue outbreak. However, its assimilation merely increased 10%. Even though people had probably realized that Madeira was at risk and that several dengue cases occurred, two erroneous interpretations could explain this 10% result. Firstly, the false belief that the ‘dengue outbreak have ended due to the eradication of the disease or the mosquito’ (alleged myths 6a/6b, Table 5). Secondly, gambler’s fallacy, the invalid belief that when something happens more frequently than normal during a period of time, the probability of happening again in the future decreases (alleged myth 3, Table 5) [14]. People who believe in these alleged myths underestimate the probability of another dengue epidemics occur in Madeira Island.

Improvements in DA1-concept, DA2-concept, LC1-concept and MB1-concept can be attributed to the “boom” of educational information transmitted during the outbreak. This information was transmitted by the news, by official reports, and most importantly by the exhaustive door-to-door campaign that was rapidly implemented in the areas where most dengue cases were being reported during the outbreak period. In the latter, trained personnel of the health-authorities entered in residential buildings and supported the residents in performing correct and extensive elimination of mosquito breeding sites inside and in the surroundings of their houses (*i.e. aegypti* source reduction). This provided a useful opportunity for residents to realize ‘the existence of larval forms/mosquitoes in their own houses’ (DA 1-concept), to ‘recognize containers that were serving as breeding sites’ (MB1-concept), to emphasize the idea that ‘domestic control could be efficient in the *A. aegypti* control’ (DA2-concept), and finally to comprehend that their ‘residential area had (indeed) vector-mosquitoes’ (LC1-concept).

In contrast to the improvement in the above stated concepts, the percentage of people who believed in ‘false mosquito breeding inducers, such as, animals or food debris’ augmented after the outbreak and thus, MB2-concept was the sole concept of which assimilation had declined after the outbreak. Female residents may have ‘erroneously indorsed *A. aegypti’*s proliferation to dirty environments’ (with food debris or animals). This assumption could be interpreted as an intuitive explanation for the appearance/establishment of the *A. aegypti* and dengue disease in the Island. As stated in psychology in the attribution theory, humans need to “attribute” causes to events which are not understood [27]. Female residents, who agreed with latter belief, and believe to live in clean households, will not feel responsible to perform domestic source reduction.

Finally, almost all the female residents agreed with the efficacy of domestic source reduction in the control of mosquitoes (CM1-concept). However, the majority still erroneously consider ‘insecticide application or flyswatter usage’ as effective measures to control mosquito population (CM2-concept). In fact, these practices are protective (i.e. can, in some manner, avoid the mosquito bite) but are not preventive (i.e. are able to control the mosquito proliferation). This mistake is determinant because people that believe in it tend to focus their efforts on these easier but less efficient practices and to disfavour the truly efficient ones, which are more difficult to implement (such as, domestic source reduction). Moreover, previous studies had shown that the local *A. aegypti* population, present in Madeira Island, was resistant to the most common insecticides, which raised questions about the reasonability of its application, even when used with protective objectives [28].

Overall, there were only three Essential Concepts that were still not considered by the majority of the studied population (LC2-concept, MB2-concept and CM2-concept). Under the assumptions of the EP-analysis, the individual minimal understanding and putative subsequent compliance to the proposed behaviour, requires the assimilation of all the ten concepts defined as ‘essential'. Consequently, the weak integration of one of these concepts by the community can compromise the usefulness of the behaviour impact campaigns. It is worth pointing out that, even though concept assimilation had generally improved after the outbreak, only 4.5% of the studied population achieved the referred ‘minimal understanding’ (EP-Score equal to ten). Consequently, there were still very few residents that are ready to engage in the proposed behaviour.

Along with the observed improvement of essential concept assimilation, myths believed by the community also changed. Even though the community is now closer to the needed ‘minimal understanding’, the task of local authorities is still difficult since after the outbreak they have to cope with new/different beliefs, following ideas such as ‘Madeira is immune to suffer a second outbreak’ (alleged myth 3 and 6).

In reality, myths could subtly persist in the community, weakening the effect of strategies aimed at behaviour changes. Therefore, an adequate monitoring of public perceptions is undoubtedly crucial to (more quickly) detect them, allowing preventive campaigns to be planned accordingly. Apart from the here observed public erroneous interpretations (probably caused by their short contact with the vector and the disease) community can provide other enriching contribution such as technical hitches in implementing proposed behaviours, pointing out messages or expressions difficult to understand, and suggesting housewives-friendly solutions [29,30].

The similarity found between paired and non-paired samples, regarding their EP-score levels supported the validity of the criteria used in the adjusted matching approach. Moreover, the observed equivalent results between the adjusted and the restricted matching procedures corroborated the validity of the geographical adjustments. Furthermore, the calculated power value supported the strength of the results albeit the apparently small size of the sample. In fact, prior sample size estimations indicated a minimal amount of 157 subjects required to fulfil the objectives of this study (as mentioned in Methods section), assuming a minimal difference (1 point) between the EP-score levels from PRE/POST-outbreak studies. However, since a difference of 2 point was observed, only 40 pairs of subjects were needed to detect it fulfilling the same objectives (S2 Table). The studied sample size was thus higher than the required to the aimed analysis. Therefore, the power associated to Wilcoxon test is also high as described in Table 6.

It is worth noticing that considering the studied sample, only women from urban areas were covered, and therefore results may not be equivalent in male subjects, rural communities or in long-term dengue regions.

In conclusion, after experiencing a dengue outbreak in Madeira Island, female community perception towards the aimed preventive engagement improved in some aspects (as intuitively expected) but also deviated in other aspects, particularly by the emergence of new myths. The most frequent myths may be used in the future to outline appropriate priority messages. Subsequent health-messages tailored according to present findings could strengthen community engagement in dengue-preventive behaviours.

Monitoring public perceptions (before/after an intervention or an outbreak) revealed a great value, not only for public health professionals but also for researchers who may be interested in investigating the complex interplay between experiences, perceptions and decision-making. Thus, lessons taken from this work can be useful not only for local authorities but also for all professionals who are engaged in dengue preparedness in endemic or epidemic countries, as well as, to those interested in strengthening tools for other behaviour-based preventable diseases.

## Supporting Information

S1 Checklist STROBE check list. (DOC)

S1 Fig Dengue outbreak incidence (2012) and PRE-outbreak and POST-outbreak study areas. Figure shows the incidence rate of the 2012 dengue outbreak (probable dengue cases per 10.000 residents). Administrative boundaries refer to ‘Municipalities’.
(TIF)

S2 Fig The EP-score median differences regarding gender from PRE-outbreak study. Figure shows the output scheme from Statistical Package for Social Sciences 19.0 (SPSS, Inc., Chicago, IL, USA) for EP-score representation by Gender in PRE-outbreak study (n = 1145).
(TIF)

S3 Fig Example of a myth. An example of how a myth can appear from partial/non-cumulative acknowledgement (not covering all essential concepts defined by the Essential Perception analysis).
(TIF)

S1 Table The EP-score median differences regarding from PRE-outbreak study. Comparison of EP-score medians, percentiles according to Gender in PRE-outbreak study total sample and respective p-value
(DOCX)

S2 Table Sample size estimation of the PRE/POST pairs required for comparison of their EP-score means. Results from Epitools’ sample size calculators for a comparison of two means using the t-test (2-tailed) [24].
(DOCX)

S3 Table Analysis of the restricted matching process Municipality adjustment. Comparisons of EP-Score medians between ‘Municipalities’ according to their Education level
(DOCX)

S4 Table Socio-demographic characterization of Funchal’s municipalities. Santa Luzia (SL), São Pedro (SP), Sé, Imaculado Coração de Maria (ICM) and Santa Maria Maior (SMM). Differences between proportions of those included in the PRE-study (SP and SL, in green) and those that were added in the POST-study (Sé, ICM and SMM in orange) are presented (in grey).
(DOCX)

S5 Table Discrepant concepts assimilation analysis POST-outbreak survey. (DOCX)
