# Supplementary figures and images for: Impact of a Dengue Outbreak Experience in the Preventive Perceptions of the Community from a Temperate Region: Madeira Island, Portugal
Source: PLoS Negl Trop Dis. 2015 Mar 13;9(3):e0003395. doi: 10.1371/journal.pntd.0003395 (PMC4388461; doi:10.1371/journal.pntd.0003395)

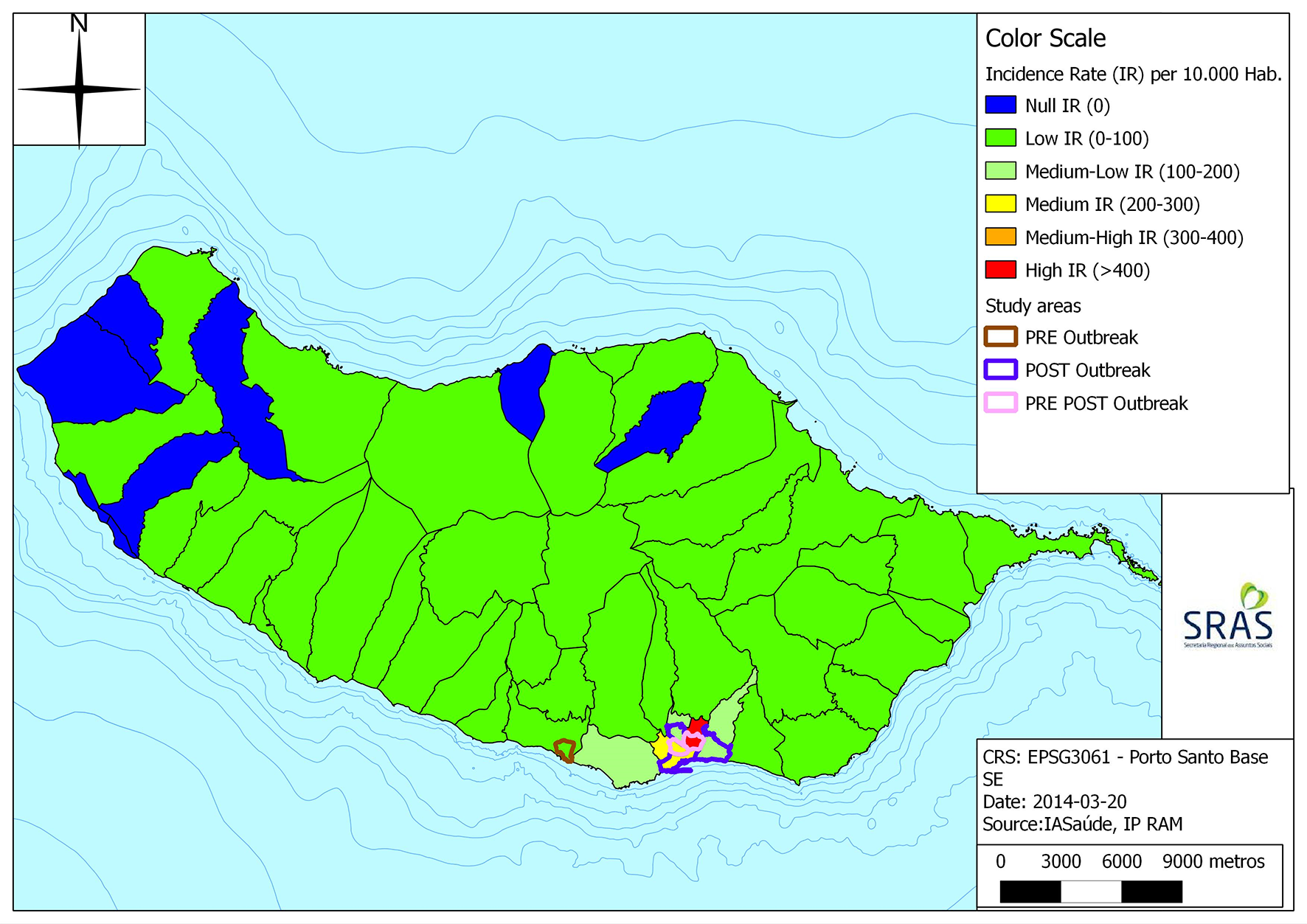

Supplement: S1 Fig — Figure shows the incidence rate of the 2012 dengue outbreak (probable dengue cases per 10.000 residents). Administrative boundaries refer to ‘Municipalities’. (TIF) [file pntd.0003395.s002.tif]

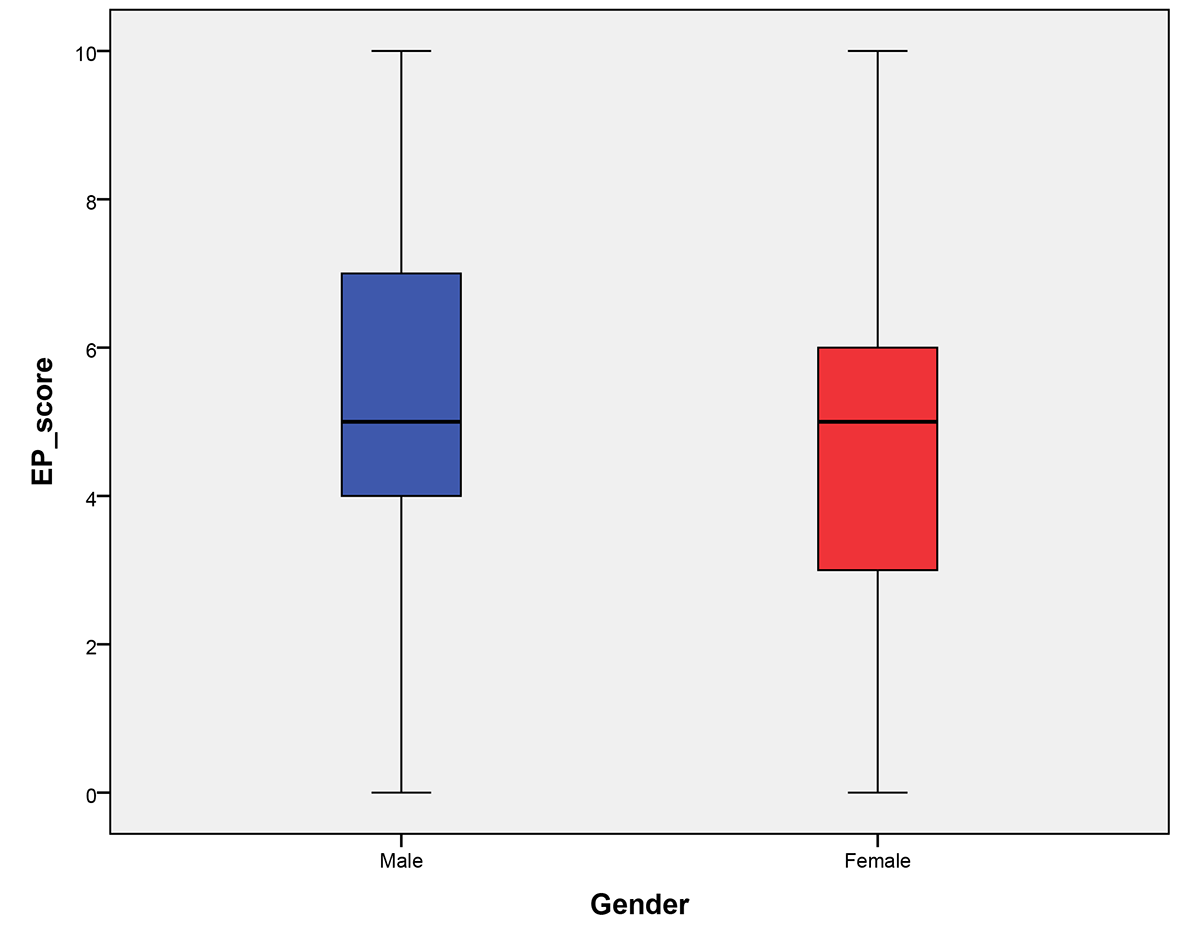

Supplement: S2 Fig — Figure shows the output scheme from Statistical Package for Social Sciences 19.0 (SPSS, Inc., Chicago, IL, USA) for EP-score representation by Gender in PRE-outbreak study (n = 1145). (TIF) [file pntd.0003395.s003.tif]

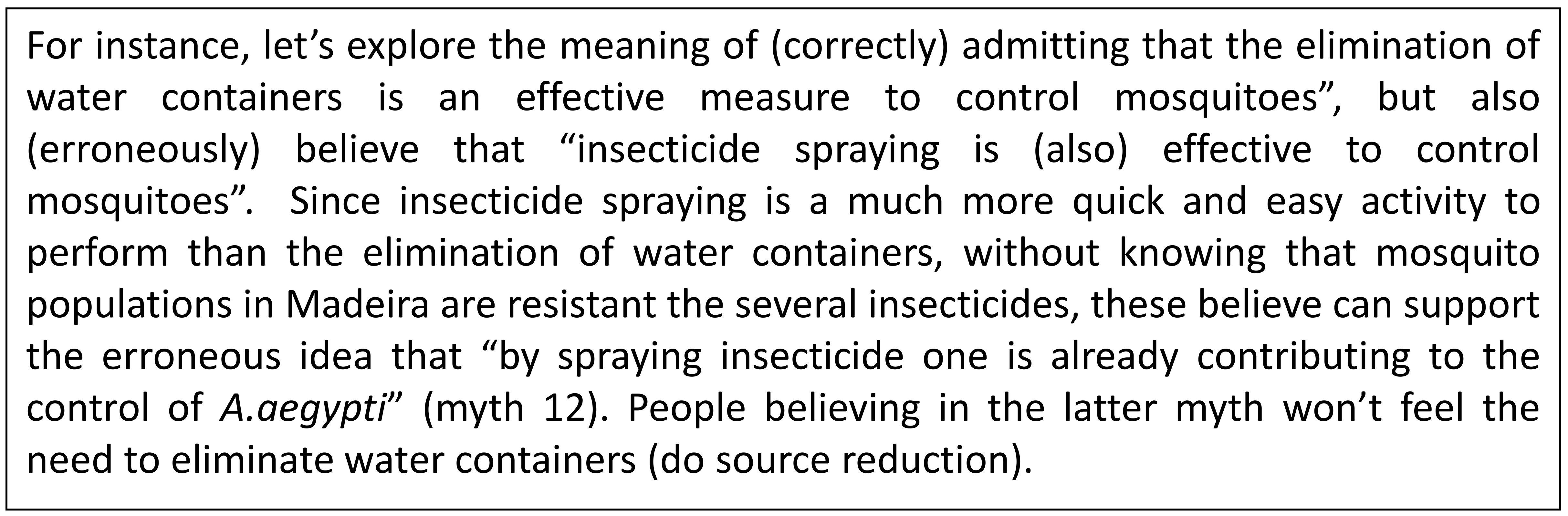

Supplement: S3 Fig — An example of how a myth can appear from partial/non-cumulative acknowledgement (not covering all essential concepts defined by the Essential Perception analysis). (TIF) [file pntd.0003395.s004.tif]
