# Supplementary material for: Impact of a Dengue Outbreak Experience in the Preventive Perceptions of the Community from a Temperate Region: Madeira Island, Portugal
Source: PLoS Negl Trop Dis. 2015 Mar 13;9(3):e0003395. doi: 10.1371/journal.pntd.0003395 (PMC4388461; doi:10.1371/journal.pntd.0003395)
Supplement: S1 Table — Comparison of EP-score medians, percentiles according to Gender in PRE-outbreak study total sample and respective p-value (DOCX) [file pntd.0003395.s005.docx]

**Table S1: The EP-score median differences regarding from PRE-outbreak study**

Comparison of EP-score medians, percentiles according to Gender in PRE-outbreak study total sample and respective p-value

|  | **EP-score median (P_25_-P_75_) ^+^** | ***p*-value** |
| --- | --- | --- |
| **Female** | 5.0 (4.0 – 7.0) | < 0.001’ |
| **Male** | 5.0 (3.0 – 6.0) |  |
| ^+^ Weighted Average method; ‘ Mann-Whitney test | | |
