# Supplementary material for: Impact of a Dengue Outbreak Experience in the Preventive Perceptions of the Community from a Temperate Region: Madeira Island, Portugal
Source: PLoS Negl Trop Dis. 2015 Mar 13;9(3):e0003395. doi: 10.1371/journal.pntd.0003395 (PMC4388461; doi:10.1371/journal.pntd.0003395)
Supplement: S2 Table — Results from Epitools’ sample size calculators for a comparison of two means using the t-test (2-tailed) [24]. (DOCX) [file pntd.0003395.s006.docx]

**Table S2: Sample size estimation of the PRE/POST pairs required for comparison of their EP-score means.** Results from Epitools’ sample size calculators for a comparison of two means using the t-test (2-tailed) [24].

|  | (1-value difference) | | | (2-value difference) | |
| --- | --- | --- | --- | --- | --- |
|  | **Inputs** | | | | |
| **Mean PRE** | | 5.0 | | | 5.0 |
| **Mean POST** | | 6.0 | | | 7.0 |
| **Variance** | | 10 | | | 10 |
| **Confidence level** | | 0.95 | | | 0.95 |
| **Power** | | 0.80 | | | 0.80 |
|  | | | **Outputs** | | |
| **Sample size**  **(per group)** | | 157 | | | 40 |
