# Supplementary material for: Impact of a Dengue Outbreak Experience in the Preventive Perceptions of the Community from a Temperate Region: Madeira Island, Portugal
Source: PLoS Negl Trop Dis. 2015 Mar 13;9(3):e0003395. doi: 10.1371/journal.pntd.0003395 (PMC4388461; doi:10.1371/journal.pntd.0003395)
Supplement: S3 Table — Comparisons of EP-Score medians between ‘Municipalities’ according to their Education level (DOCX) [file pntd.0003395.s007.docx]

**Table S3: Analysis of the restricted matching process Municipality adjustment**

Comparisons of EP-Score medians between ‘Municipalities’ according to their Education level

|  | **Municipality** | |  |
| --- | --- | --- | --- |
|  | **Santa Luzia** | **São Pedro** | ***p*-value** |
| **EP-score median (P_25_-P_75_) ^+^ (Education Level =1)** | 5.00 (2.25-5.00) | 3.00 (1.00-5.00) | 0.360’ |
| **EP-score median (P_25_-P_75_) ^+^ (Education Level =2)** | 4.00 (3.00-5.00) | 4.00 (2.25-5.00) | 0.186’ |
| **EP-score median (P_25_-P_75_) ^+^ (Education Level =3)** | 5.00 (4.00-7.00) | 5.00 (4.00-6.00) | 0.824’ |
| **EP-score median (P_25_-P_75_) ^+^ (Education Level =4)** | 6.00 (5.00-7.00) | 5.00 (4.00-7.00) | 0.178’ |
| **EP-score median (P_25_-P_75_) ^+^ (Education Level =5)** | 7.00 (6.00-8.00) | 7.00 (5.50-7.00) | 0.844’ |
| ^+^ Weighted Average method ; ‘ Mann-Whitney test | | | |
