# Supplementary material for: Impact of a Dengue Outbreak Experience in the Preventive Perceptions of the Community from a Temperate Region: Madeira Island, Portugal
Source: PLoS Negl Trop Dis. 2015 Mar 13;9(3):e0003395. doi: 10.1371/journal.pntd.0003395 (PMC4388461; doi:10.1371/journal.pntd.0003395)
Supplement: S4 Table — Santa Luzia (SL), São Pedro (SP), Sé, Imaculado Coração de Maria (ICM) and Santa Maria Maior (SMM). Differences between proportions of those included in the PRE-study (SP and SL, in green) and those that were added in the POST-study (Sé, ICM and SMM in orange) are presented (in grey). (DOCX) [file pntd.0003395.s008.docx]

**Table S4: Socio-demographic characterization of Funchal’s municipalities**

Santa Luzia (SL), São Pedro (SP), Sé, Imaculado Coração de Maria (ICM) and Santa Maria Maior (SMM). Differences between proportions of those included in the PRE-study (SP and SL, in green) and those that were added in the POST-study (Sé, ICM and SMM in orange) are presented (in grey).

|  | **Female residents** | | **Female**  **15-19 years** | | **Female**  **20-24 years** | | **Female**  **20-64 years** | | **Female**  **25-64 years** | | **Female over 64 years** | |
| --- | --- | --- | --- | --- | --- | --- | --- | --- | --- | --- | --- | --- |
| **SL** | 55.7 | | 2.5 | | 2.4 | | 31.6 | | 29.2 | | 15.3 | |
| **SP** | 55.4 | | 2.4 | | 2.6 | | 32.6 | | 30.0 | | 13.0 | |
| **Mean SL,SP (A)** | **55.6** | | **2.5** | | **2.5** | | **32.0** | | **29.5** | | **14.5** | |
| **Sé** | 58.2 | | 2.8 | | 2.1 | | 32.5 | | 30.4 | | 16.7 | |
| **ICM** | 54.2 | | 5.1 | | 5.2 | | 34.6 | | 31.6 | | 12.4 | |
| **SMM** | 54.3 | | 2.5 | | 2.8 | | 31.3 | | 28.4 | | 14.6 | |
| **Mean Sé,ICM,SMM (B)** | **55.1** | | **3.2** | | **3.3** | | **32.4** | | **29.6** | | **14.4** | |
|  |  | |  | |  | |  | |  | |  | |
| Difference (A – B) | -0.5 | | 0.7 | | 0.8 | | 0.4 | | 0.1 | | 0.0 | |
|  |  | |  | |  | |  | |  | |  | |
| **%** | **Never studied** | **4^th^grade** | | **6^th^grade** | | **9^th^grade** | | **12^th^grade** | | **Post-secondary school** | | **Degree** |
| **SL** | 2.1 | 20.0 | | 11.2 | | 16.9 | | 17.1 | | 0.9 | | 20.5 |
| **SP** | 3.5 | 24.6 | | 11.9 | | 16.4 | | 13.5 | | 1.4 | | 15.5 |
| **Mean SL,SP (A)** | **2.6** | **21.7** | | **11.4** | | **16.8** | | **15.8** | | **1.1** | | **18.6** |
| **Sé** | 2.1 | 17.7 | | 9.5 | | 15.7 | | 15.6 | | 1.0 | | 27.3 |
| **ICM** | 7.3 | 26.6 | | 15.2 | | 17.7 | | 14.9 | | 3.4 | | 15.4 |
| **SMM** | 3.3 | 23.6 | | 12.8 | | 16.6 | | 15.6 | | 1.0 | | 16.2 |
| **Mean Sé,ICM,SMM (B)** | **4.1** | **23.2** | | **12.8** | | **16.7** | | **15.4** | | **1.6** | | **18.2** |
|  |  |  | |  | |  | |  | |  | |  |
| Difference (A – B) | 1.5 | 1.5 | | 1.3 | | -0.1 | | -0.3 | | 0.5 | | -0.5 |
