# Supplementary material for: Impact of a Dengue Outbreak Experience in the Preventive Perceptions of the Community from a Temperate Region: Madeira Island, Portugal
Source: PLoS Negl Trop Dis. 2015 Mar 13;9(3):e0003395. doi: 10.1371/journal.pntd.0003395 (PMC4388461; doi:10.1371/journal.pntd.0003395)
Supplement: S5 Table — (DOCX) [file pntd.0003395.s009.docx]

Table S5: Discrepant concepts assimilation analysis POST-outbreak survey

| **Concepts acknowledgement comparison** | | | **Analysis of Topic’s Understanding** |  | **Community Understanding / Alleged myths** |
| --- | --- | --- | --- | --- | --- |
|  | |  |  |  |  |
| **MI1-(concept)** | **MI2- (concept)** |  | **Medical Importance** | | |
|  |  | 72.2 **%**  (62 ind.) | Residents admitted that mosquitoes transmit diseases such as ‘Dengue’ (55·6%) or other mosquito-borne diseases. |  | Residents seemed to understand the real medical importance of mosquitoes and, thus the relevance of being involved in the *aegypti*-control. |
|  | X | 22.2 **%**  (20 ind.) | Even though admitting that mosquitoes can transmit diseases, these residents did not know what kind of diseases mosquitoes transmit. Some residents erroneously referred ‘allergies’ or other false clinical consequences such us ‘fever’. |  | These residents were not aware of the relevance of being involved in the *aegypti*-control.  **Alleged Myth 2: “Mosquitoes only cause mild clinical consequences such as allergies, fever, etc.”** |
| X |  | Not observed | | | |
| X | X | 5. 6**%**  (5 ind.) | Residents did not know that mosquitoes can transmit diseases |  | Residents did not understand the medical importance of mosquitoes.  **Alleged Myth 1: “Mosquitoes do not transmit diseases”** |
| **LC1** | **LC2** |  | **Local Context** | | |
|  |  | 36.7 **%**  (33 ind.) | Residents recognized that there were mosquitoes that transmit diseases in their residential area, and, also, that there was a risk of a dengue outbreak in Madeira. |  | Residents seemed to understand the local risk they are submitted and, thus the urgency of being involved in the *aegypti*-control. |
| - X | | 37.8 **%**  (34 ind.) | Residents recognized the presence of mosquitoes that transmit diseases in their residential area; however they believed that a dengue outbreak will not re-emerge in the island. Eventually some residents could think that Madeira is now “protected”. Since a dengue outbreak has just occurred, there is a current very low probability of another dengue outbreak to emerge (gambler’s fallacy). Other possibility is that some residents could think that the end of the outbreak occurred when the mosquito/disease was eradicated from the island, and thus, now it won’t occur anymore. |  | These residents were not aware of the urgency of being involved in the *aegypti*-control.  **Alleged myth 3: “‘Dengue will not occur again in Madeira, it is very not likely‘”;** |
| X |  | 10.0 **%**  (9 ind.) | Residents did not recognize the presence of mosquitoes that can transmit diseases in their residential area; but admitted that a dengue outbreak can emerge in the island. These residents did not have a correct notion of the *aegypti’s* distribution area. |  | Residents did not understand the risk they are subjected to and neither the urgency of being involved in the *aegypti*-control.  **Alleged myths 4 and 5: (i) -“Since I do not feel the byte, I am not at risk of being bitten/infected”. (ii) - “Mosquitoes are allocated in a specific area and are not able to spread to my municipality”;** |
| X | X | 15.6 **%**  (14 ind.) | Residents did not recognize mosquitoes that transmit diseases in their residential area neither the possibility of a dengue outbreak in the island. |  | Residents did not understand the risk they are subjected to neither the urgency of being involved in the *aegypti*-control.  **Alleged Myth 6: “Dengue/*A. aegypti* was, finally, eradicated”.** |
| **DA1** | **DA2** | **%** | **Domestic Attribute** | | |
|  |  | 55.6 **%**  (50 ind.) | Residents know that mosquitoes can breed inside houses and recognized that domestic *aegypti-*control do have impact in the reduction of *aegypti*-population. |  | Residents seemed to understand the domestic attribute of the *aegypti*-control and, thus why community is the key intervenient in the *aegypti*-control. |
|  | X | 13.3 **%**  (12 ind.) | Residents know that mosquitoes can breed inside houses but they did not believe that the domestic *aegypti-*control have impact in the reduction of the *aegypti*’s population. They probably believed that other intervenients have much more impact in the reduction of the *aegypti*’s population. |  | Residents did not understand the domestic attribute of the *aegypti*-control, neither why community is the key intervenient in the *aegypti-*control.  **Alleged Myth 7: “Local health authorities are the key intervenient in the control of mosquitoes”.** |
| X |  | 18.9 **%**  (17 ind.) | Mosquitoes cannot breed inside houses but domestic *aegypti-*control does have impact in the reduction of *aegypti-*population in the neighborhood. Those respondents believed in their role in domestic *aegypti*-control but did not understood why that control has an impact, probably by avoided them to enter in the house. |  | Residents did not understand the domestic attribute of the *aegypti*-control, neither why community is the key intervenient in the *aegypti-*control.  **Alleged Myth 8: “Other protective measures can control mosquitoes”.** |
| X | X | 12.2 **%**  (11 ind.) | Residents do not know that mosquitoes can breed inside houses, neither that their involvement have an impact in the control of mosquitoes. |  | Residents did not understand the domestic attribute of the *aegypti*-control, neither why community is the key intervenient in the *aegypti-*control.  **Alleged Myth 9: “I am not/Community is not an intervenient in the aegypti-control”**. |
| **MB1** | **MB2** | **%** | **Mosquito Breeding** | | |
|  |  | 35.6 **%**  (32 ind.) | Residents only identified water-containers (and not other false issues) as mosquitoes’ breeding inducers. |  | Residents seemed to understand where do mosquito breed and, thus the need of the *aegypti*-control activities. |
|  | X | 61.1 **%**  (55 ind.) | Residents identified water-containers but also other false issues (food debris and pets) as mosquitoes’ breeding inducers These residents did not comprehend what lead to the breeding of new mosquitoes and, thus did not understand the proposed measures to control them. |  | Residents seemed to not understand where mosquitoes breed and neither the need of the *aegypti*-control activities.  **Alleged Myths 10 and 11: “Clean houses or houses without pets/animals do not have mosquitoes” or “Clean people did not need to be involved in mosquito control”.** |
| X |  | 1.1 %  (1 ind.) | Residents did not identify water-containers neither other false issues (food debris and pets) as mosquitoes’ breeding inducers. These residents did not know where do mosquitoes breed or believe in other false breeding sites. |  | Residents seemed to not understand where mosquitoes breed and neither the need of the *aegypti*-control activities. |
| X | X | 2.2 %  (2 ind.) | Residents did not identify water-containers but did identify other false issues (food debris and pets) as mosquitoes’ breeding inducers |  | Residents are completely mistaken regarding mosquitoes breeding and, thus did not understand the need of the *aegypti*-control activities.  **Alleged Myths 10 and 11: “Clean houses or houses without pets/animals do not have mosquitoes” or “Clean people did not need to be involved in mosquito control”.** |
| **CM1** | **CM2** | **%** | **Control Measures** | | |
|  |  | 36.7 %  (33 ind.) | Residents only recognized water-containers removal (and not other false measures) as “effective to control mosquitoes” |  | Residents seemed to recognize effective control measures and, thus understand how the domestic *aegypti*-control should be done. |
|  | X | 61·1 %  (55 ind.) | Residents recognized water-containers removal and also other false measures (such as insecticide indoor application and flyswatter use) as “effective to control mosquitoes” |  | Residents seemed to not be focused on effective control measures and, thus did not understand how the domestic *aegypti*-control should be done.  **Alleged Myth 12: “By using protective measures (such as insecticides or the flyswatter), I am already contributing to control the *aegypti*-mosquito”** |
| X |  | 1.1 %  (1 ind.) | Residents did not recognize water-containers removal neither other false measures (such as insecticide indoor application and flyswatter use) as “effective to control mosquitoes”. These residents did not know how to control mosquitoes. |  | Residents not recognized effective control measures and, thus did not understand how domestic *aegypti*-control should be done. |
| X | X | 1.1  (1 ind.) | Residents recognized water-containers removal and also other false measures (such as insecticide indoor application and flyswatter use) as “effective to control mosquitoes” |  | Residents seemed to not be focused on effective control measures and thus did not understand how the domestic aegypti-control should be done.  **Alleged Myth 12: “By using protective measures (such as insecticides or the flyswatter), I am already contributing to control the *aegypti*-mosquito”** |
